# Thromboxane A_2_ Regulates CXCL1 and CXCL8 Chemokine Expression in the Nasal Mucosa–Derived Fibroblasts of Chronic Rhinosinusitis Patients

**DOI:** 10.1371/journal.pone.0158438

**Published:** 2016-06-28

**Authors:** Yih-Jeng Tsai, Sheng-Po Hao, Chih-Li Chen, Wen-Bin Wu

**Affiliations:** 1 Department of Otolaryngology Head and Neck Surgery, Shin Kong Wu Ho-Su Memorial Hospital, Taipei, Taiwan; 2 Department of Chemistry, Graduate Institute of Applied Science and Engineering, College of Science and Engineering, Fu-Jen Catholic University, Taipei, Taiwan; 3 Graduate Institute of Basic Medicine, Fu-Jen Catholic University, Taipei, Taiwan; 4 School of Medicine, Fu-Jen Catholic University, Taipei, Taiwan; Chang Gung University, TAIWAN

## Abstract

**Background:**

Chronic rhinosinusitis without nasal polyps (CRSsNP) is a common chronic disease and the etiology remains unclear. Thromboxane A_2_ (TXA_2_) participates in platelet aggregation and tissue inflammation. In this study, the CXCL1/8 chemokine and TXA_2_-TP receptor expression in the CRSsNP mucosa was investigated.

**Experimental Approach:**

Immunohistochemistry, chemokine release assay by ELISA, RT-PCR, Real-time PCR, Western blotting, pharmacological and siRNA knockdown analysis were applied in the CRSsNP tissue specimen and cultured nasal mucosa-derived fibroblasts.

**Results:**

The immunohistochemistry results indicated that CXCL1 and CXCL8 were highly expressed in the CRSsNP mucosa compared with the controls; however, the TP receptors were expressed in both mucosa. Therefore, U46619 and IBOP, a TXA_2_ analog and TP agonist, were used to explore the role of TP activation in CXCL1/8 expression; both of these induced CXCL1/8 mRNA and protein expression in CRSsNP mucosa-derived fibroblasts. U46619 phosphorylated PI-3K, cyclic AMP (cAMP)/PKA, PKC, and cAMP response element (CREB). Activation of cAMP/PKA, PKC, and CREB was the major pathway for *cxcl1/8* gene transcription. Pharmacological and siRNA knockdown analyses revealed that activation of cAMP/PKA and PKCμ/PKD pathways were required for CREB phosphorylation and PKA/C crosstalked with the PI-3K pathway.

**Conclusion and Implications:**

Our study provides the first evidence for abundant TP receptor and CXCL1/8 expression in human CRSsNP mucosa and for TXA_2_ stimulation inducing CXCL1/8 expression in nasal fibroblasts primarily through TP receptor, cAMP/PKA, PKCμ/PKD, and CREB-related pathways.

## Introduction

Chronic rhinosinusitis (CRS), one of the most common chronic airway inflammatory diseases, is common in all healthcare settings. CRS severely influences the quality of life. In addition, it results in substantial annual healthcare expenditure [1]. CRS is a multifactorial inflammatory disease of the nasal cavity and paranasal sinuses. Although its pathophysiology remains unclear, increasing evidence reveals that in addition to infection and obstruction, immunologic inflammatory responses are crucial in the pathophysiology of CRS [2].

Proinflammatory cytokines and arachidonic acid metabolites, including prostaglandins (PGs), leukotrienes, and thromboxanes (TXs), are crucial for the existing mucosal inflammatory reactions in many inflammatory conditions, such as allergic rhinitis and chronic rhinosinusitis, being activating factors for epithelial cells, endothelial cells, T lymphocytes, and other cell types [3,4]. CRS is not only a consequence of infection but also a chronic inflammatory condition that can become an acute clinical event through tissue remodeling and invasion [2]. Pathological studies have observed a defined series of changes in the nasal mucosa and revealed that blood-derived inflammatory cells, particularly T cells, eosinophils, neutrophils, and macrophages, are important during chronic inflammation [5]. Despite great advances in the understanding their pathophysiology [6,7], the accurate etiology of chronic inflammatory conditions of the nose and sinuses is still essentially unclear. Remodeling is a critical aspect of wound repair in organs and is a dynamic process resulting in both extracellular matrix production and degradation. CRS can be categorized into CRS with nasal polyps (CRSwNP) and CRS without nasal polyps (CRSsNP) according to different remodeling pattern and tissue inflammation profiles [8,9]. CRSsNP accounts for about 60% cases of CRS and CRSwNP causes approximately 20%–33% [10].

Chemokines (chemotactic cytokines) are a group of small (8–14 kDa), heparin-binding proteins that regulate the movement of circulating leukocytes to the inflammation sites [11,12]. Concerning the inflammation, increasing evidence reveals that chemokine levels potentially affect the progression of airway inflammation [13,14]. CXC chemokines, one of the subfamily of chemokines, participate in many inflammatory process. For example, CXCL1 and CXCL8, are chiefly promoters and contribute to many tissue inflammation and process of angiogenesis [6]. CXCL1 and CXCL8 generally regulate the movement of circulating leukocytes to inflammation or injury sites through binding of leukocyte receptor, CXCR1 and CXCR2 [15,16]. Rudack et al., showed that after protease-activated receptor-2 (PAR-2) stimulation, the CXCL1 and CXCL8 can be synthesized by activating nuclear factor-kappa B (NF-κB) signaling in their CRS study [7].

PGs and TXs are arachidonic acid metabolites involved in platelet aggregation and cell inflammation [17]. TXA_2_ is a thromboxane predominantly produced by activated platelets and has prothrombotic properties. Moreover, it is a vasoconstrictor particularly important during tissue injury and inflammation. TXA_2_ participates in cutaneous immune response through TP receptor (TXA_2_ receptor) and subsequent Gs protein-cAMP-PKA signaling or Gq protein-phospholipase C-PKC pathway. This might be important in atopic dermatitis and pruritus [18]. Pe´rez-Novo et al., revealed that PGD_2_ was increased in CRSwNP tissue and promote the migration of Th2 cells through a CRTH2 dependent mechanism [19]. However, their role in the pathogenesis of CRSsNP remains unknown. Therefore, in this study, we examined TP receptor and CXC chemokine expression in the CRSsNP mucosa and the effects of TXA_2_ on chemokine release in the nasal fibroblasts derived from CRSsNP mucosa and the underlying mechanism.

## Materials and Methods

### Materials

U46619 (a TXA_2_ analog), [1S-[1α,2α(Z),3β(1E,3S*),4α]]-7-[3-[3-hydroxy-4-(4-iodophenoxy)-1-butenyl]-7-oxabicyclo[2.2.1]hept-2-yl]-5-heptenoic acid (IBOP; a TP receptor agonist), and SQ29548 were purchased from Cayman Chemicals (Ann Arbor, Michigan, USA). LY294002, and actinomycin D (Act D) were purchased from Sigma-Aldrich Chemical Corp (St Louis, MO, USA). Go6983 (Tocris Cookson Ltd., Bristol, BS, UK), H-89, and GF109203X were from Biomol (Farmingdale, NY, USA). The antibodies (Abs) generated against phospho-ERK1/2 were obtained from Santa Cruz Biotechnology (Dallas, TX, USA). The Abs raised against PKA/C substrates, cyclic AMP (cAMP) response element (CREB), phospho-CREB, and PKC isoforms were obtained from Cell Signaling Technology, Inc. (Danvers, MA, USA). The Ab against ERK1/2 was purchased from R&D systems, Inc. (Minneapolis, MN, USA). The Ab raised against α-tubulin was purchased from EMD Millipore (Billerica, MA, USA).

### Patient Recruitment

This study was approved (Permission No:20130812R) by the Ethics Committee of Shin Kong Wu Ho-Su Memorial Hospital, Taipei, Taiwan, and written informed consent were obtained from the patients. In total, 14 patients with CRSsNP were recruited, and 13 patients came for correcting nasal septal deviations were recruited as the control group. CRSsNP was diagnosed on the basis of patient history, local findings from anterior rhinoscopy, nasal endoscopy, and sinus computed tomography. No patient had a history of allergy, asthma, or aspirin sensitivity, and none had been treated with either oral or topical antiallergic agent or steroid for at least 2 months. In the CRSsNP group, the ethmoidal mucosa and the mucosa around the osteomeatal complex were retrieved during functional endoscopic sinus surgery, and mucosal samples of the inferior turbinate were attained from patients who had undergone septoplasty in the control group.

### Isolation of Nasal Mucosa–Derived Fibroblasts (NMDFs)

NMDFs were prepared from the nasal mucosa of those who were diagnosed as CRSsNP as previously described by our lab [20]. Briefly, the nasal tissues were minced into fragments. Then the fragments were plated in 6-well culture dishes in Dulbecco Modified Eagle’s Medium supplemented with 100 U/mL penicillin, 100 μg/mL streptomycin, and 250 ng/mL Fungizone (Thermo Fisher Scientific, Waltham, MA, USA) and 10% fetal bovine serum (FBS). The fibroblasts were isolated from the tissue fragments through adhesion and migration on a plastic culture plate and were identified as fibroblasts with positive staining for the presence of vimentin (Santa Cruz Biotechnology).

### ELISA Measurement of CXCL1 and CXCL18 Secreted in the Culture Medium

The human CXCL1 and CXCL8 ELISA development kit (R&D Systems, Inc., MN, USA) was applied to determine the CXCL1 and CXCL8 in the culture medium according to the procedure described by the manufacturer. In brief, the culture medium was collected and centrifuged, and the CXCL1/CXCL8 released into the culture medium was measured at 412 nm. The concentration of CXCL1 and CXCL8 in the NMDF culture medium were calculated from the standard curve.

### Reverse Transcription-Polymerase Chain Reaction (RT-PCR) Analysis and Real-Time PCR Analysis of CXCL1 and CXCL8 mRNA Expression

Oligonucleotide PCR primers targeting human CXCL1, CXCL8, and β-actin were synthesized using MDBio Inc. (Taipei, Taiwan; Table 1). After the total RNA was extracted by Trizol reagent (Thermo Fisher Scientific), reverse transcription was performed using Superscript III First-Strand Synthesis System (Thermo Fisher Scientific). The RT-PCR analyses of CXC chemokines and β-actin were performed as previously described [21], and the products were identified through electrophoresis on 2% agarose gel. The CXCL1 and CXCL8 mRNA level were also analyzed by real-time PCR with the TaqMan gene expression assay system (Life Technologies, Applied Biosystems, Grand Island, NY, USA), using primers/probe sets Hs.708652 for human CXCL1, Hs.174103 for human CXCL8 and Hs.520640 for human β-actin (as a control). PCRs were performed using a 7500 Real-Time PCR System (Life Technologies, Applied Biosystems, Grand Island, NY, USA). Relative gene expression was determined by the ΔΔCt method, where Ct was the threshold cycle. All experiments were performed in duplicate or triplicate.

**Table 1:** Primers for RT-PCR analysis.

Gene	Forward primer(5’-3’)	Reverse primer (5’-3’)
*cxcl1*	GCCCAAACCGAAGTCATAGCC	ATCCGCCAGCCTCTATCACA
*cxcl8*	TTTGCCAAGGAGTGCTAAAG	GCATCTGGCAACCCTACAAC
*β-actin*	ATCATGTTTGAGACCTTCAA	CATCTCTTGCTCGAAGTCCA

### CXCL1 Reporter Luciferase Assay

A CXCL1 luciferase reporter assay was performed as previously described [21]. Cells in 12-well culture plates at approximately 80% confluence were transfected with 0.75 μg of total DNA using GenJet^TM^ In Vitro DNA Transfection Reagent (SignaGen Lab, Rockville, MD, USA) for 18 h in the medium according to manufacturer protocol. All transient transfections had 0.75 μg of CXCL1 reporter construct and the pSV-α-galactosidase control vector (Promega). CXCL1 reporter firefly luciferase values were obtained by analyzing 1 mL of purified cell extract according to manufacturer instructions using a Wallac Victor 3 1420 multilabel counter (Perkin Elmer, Turku, Finland). β-Gal activity was used as the internal control and was determined using o-nitrophenyl-β-d-galactopyranoside as the substrate (Green and Sambrook, 2012). For the pCREB-Luc reporter assay, the cells were transfected with pCREB-Luc (a reporter construct containing CREB [Signosis Inc., Sunnyvale, CA, USA]). Reporter transfection was performed by seeding cells (1.5 × 10^5^ cells/mL) in 12-well culture plates; the cells were subsequently transfected with 0.75 μg of total DNA using GenJet^TM^ In Vitro DNA Transfection Reagent for 18 h in the medium according to manufacturer protocol. Following transfection, the cells were washed once with endotoxin-free medium and allowed to grow for 24 h in complete medium containing antibiotics. The reporter luciferase activities of cell extracts were measured as described earlier in the text.

### Cell Lysate Preparation and Western Blot Analysis

The cellular lysates were prepared as described previously [22]. Total proteins were separated through electrophoresis using SDS-polyacrylamide gels, electroblotted to PVDF membranes, and subsequently probed using a primary Ab. The immunoblots were developed using Immobilon Western Chemiluminescent HRP Substrate (EMD Millipore Corporation, Billerica, MA, USA). For some experiments, the membranes were stripped with striping buffer (62.5 mM Tris-HCl, pH 6.7, 2% SDS and 100 mM β-mercaptoethanol), washed, and reprobed with Abs to examine the levels of α-tubulin or the corresponding total proteins and were subsequently developed as described earlier in the text.

### Expression Patterns of Chemokines and TP Receptor in Control and CRS Biopsies

The chemokines and TP receptor expression in the control and CRS biopsied tissues were determined through immunohistochemistry. Briefly, tissue sections were deparaffinized, and the slides were hydrated in graded ethanol before use. The sections were subsequently washed in Tris-buffered saline (TBS containing 1% CaCl_2_), immersed in sodium citrate buffer (pH 6.0), and heated on a water bath for 20 min. The slides were incubated at 4°C overnight with primary Ab specific for CXCL1 (LifeSpan BioSciences, Inc., Seattle, WA, USA), CXCL8 (R&D systems, Inc., MN, USA), and TP receptor (LifeSpan BioSciences, Inc., Seattle, WA, USA) after blocking with a buffer containing 10% FBS. Secondary antibodies were used at a dilution of 1:250. After additional washing, the slides were stained with one-step 3-amino-9-ethylcarbazole (AEC; Biogenex) for 5–30 min. Sections were counterstained in hematoxylin for 20–40s, washed with tap water, and mounted with 100% glycerol.

### ELISA Measurement of Intracellular cAMP Production

Intracellular cAMP production was measured using Cayman cAMP EIA kit (Cayman Chemical Company, Ann Arbor, MI) according to manufacturer’s instruction. Briefly, the cells were treated with U46619 for 10 min and collected; the samples were acetylated by adding acetic acid anhydride. The intracellular cAMP was measured through the acetylcholine esterase competitive enzyme immunoassay, which yielded a yellowish product that absorbs strongly at 420 nm. The intensity of this color is proportional to the amount of the cAMP tracer bound to the well, which is inversely proportional to the amount of free cAMP present in the well during incubation. The acetylated cAMP concentrations in cell lysates were calculated using the standard curve.

### siRNA Transfection

SiGenome SMARTpool control, PKC, and CREB siRNAs were purchased from Dharmacon RNAi Technologies (Thermo Fisher Scientific). The transfection was performed according to manufacturer’s protocol but with some modifications. The cells were seeded in 6-well plates and incubated overnight in complete medium. The cell cultures were transfected with control, PKC and CREB siRNAs (150 nM) for 3 days using the DharmaFECT transfection reagent. The cells and media were collected after treatment with U46619 and were analyzed through Western blotting and ELISA.

### Statistical Analysis

The data are expressed as the mean ± standard error mean (SEM). The means of data from the 2 groups were compared using unpaired two-tailed Student *t* tests.

## Results

### CXCL1 and CXCL8 Expression Patterns in the Mucosa of Control (NSD) and CRSsNP Specimens

**Fig 1:**
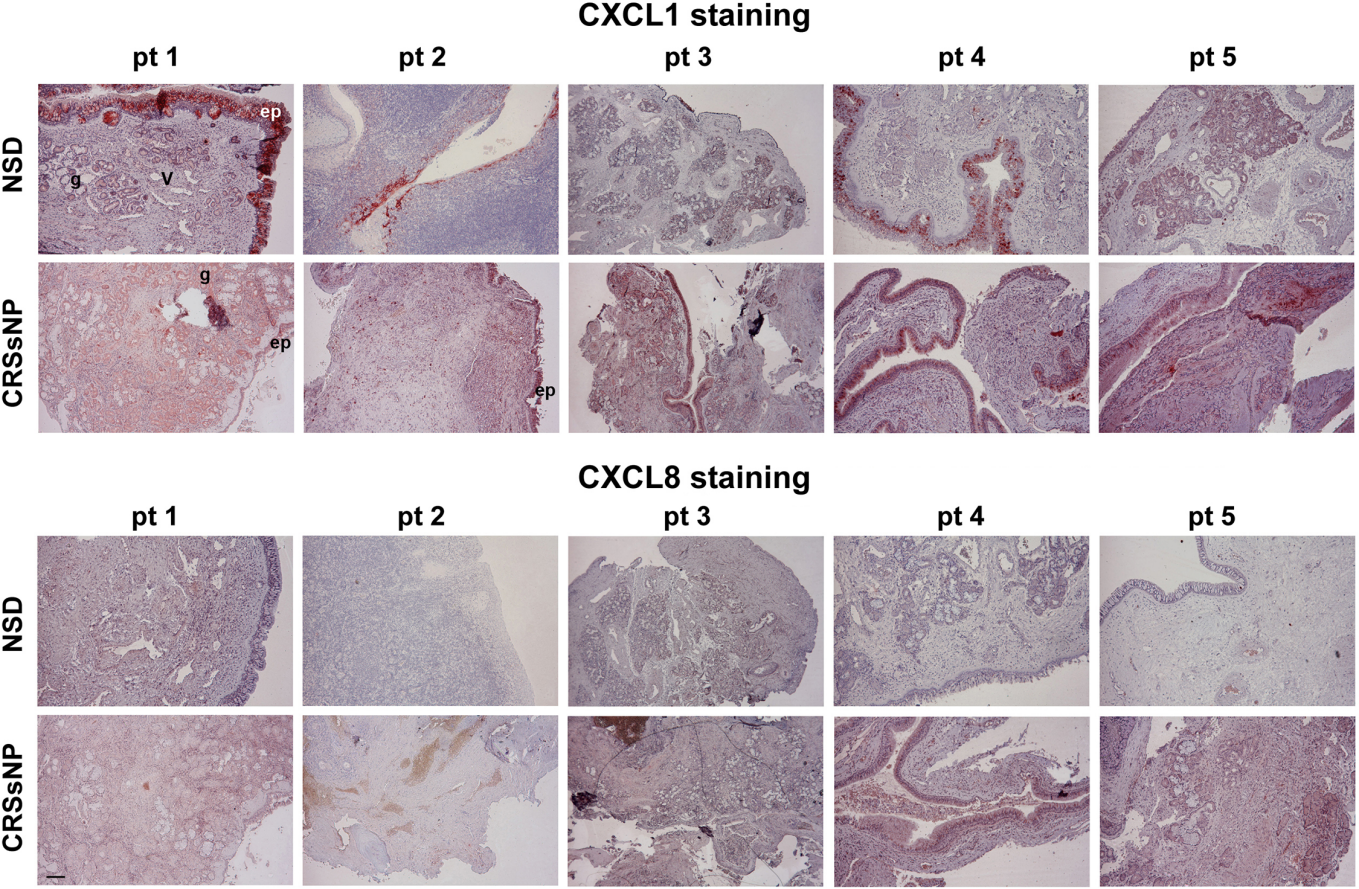
CXCL1 and CXCL8 expression in the mucosa specimens. (A) Immunohistochemical staining of CXCL1 and CXCL8 distribution in the control and CRSsNP nasal mucosa. The inferior turbinates (control) and CRSsNP nasal mucosa from 5 individual patients (pt) were stained with anti-CXCL1 and CXCL8 Ab. Immunoreactivity for CXCL1 and CXCL8 (deep-red staining) was stronger in the submucosal stroma of CRSsNP specimens than in the nasal septal deviation controls. ep: epithelial cells; v: blood vessels; g: submucosal glands. Scale bar = 100 μm.

The immunohistochemistry of the specimens was performed using the Ab generated against CXCL1 and CXCL8. Fig 1 shows the representative mucosa specimens from 5 control and CRSsNP patients. CXCL1 and CXCL8 (deep-red color staining) were more strongly expressed in the submucosal stroma, vessel wall, and glands of the CRSsNP specimens compared with specimens with nasal septal deviation (NSD, controls).

### Role of Thromboxane A_2_ (TXA_2_) in CXCL1 and CXCL8 Protein Secretion and mRNA Levels

**Fig 2:**
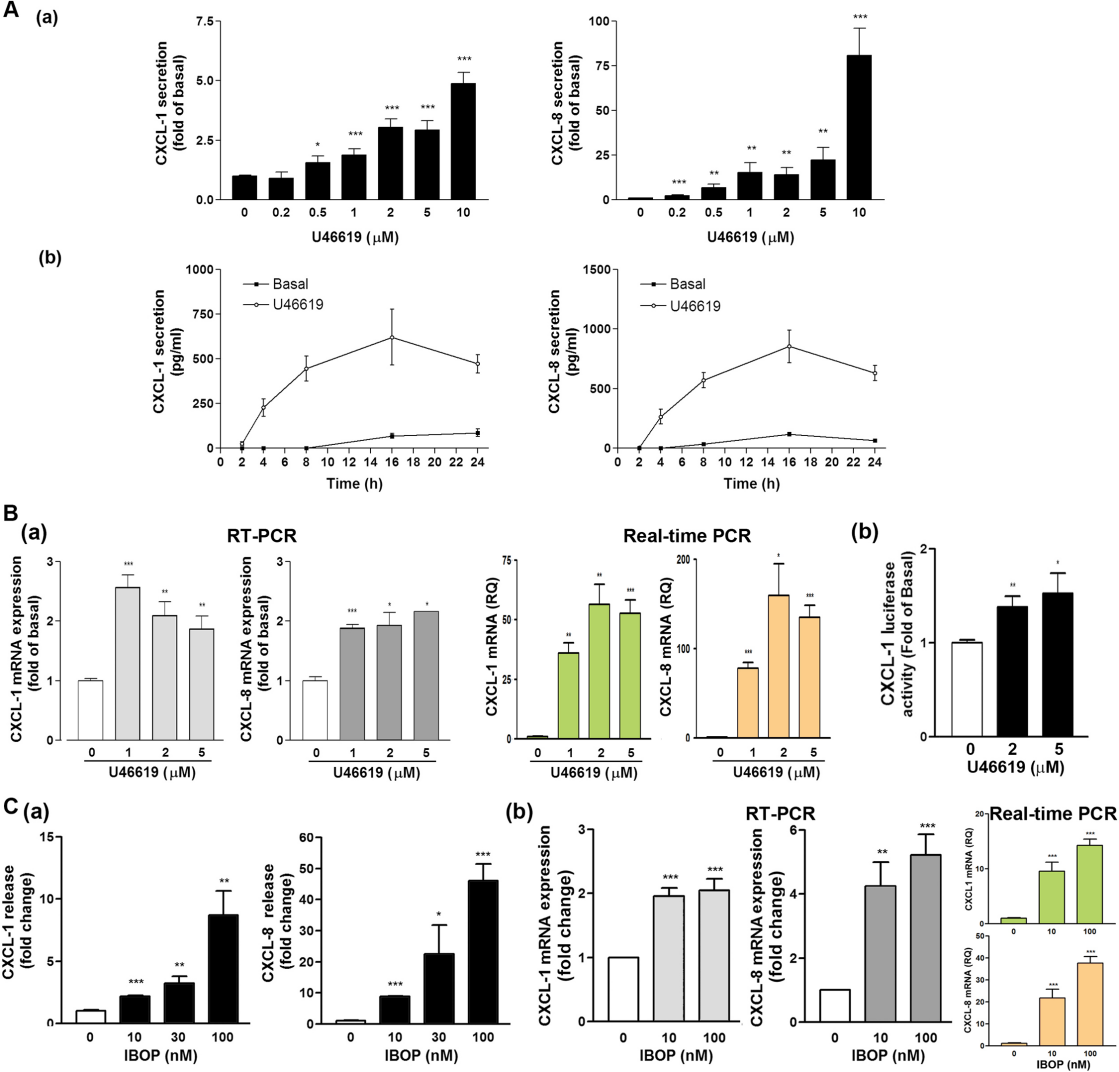
Effect of U46619 and IBOP on CXCL1 and CXCL8 expression. (A) The NMDFs were treated with (a) the indicated concentrations of U46619 for 16 h or (b) U46619 (2 μM) for the indicated time intervals. CXCL1 and CXCL8 in the culture media were analyzed through ELISA. (B) (a) NMDFs were treated with the indicated concentrations of U46619 for 4 h. At the end of the incubation, total RNA was extracted, and the expression of CXC chemokines and GAPDH mRNA was analyzed through RT-PCR and real-time PCR (n = 3).*P < 0.05, **P < 0.01 versus basal (without stimulation). (b) The NMDFs were transfected with CXCL1 promoter luc reporter plasmid for 48 h and followed by U46619 stimulation. The CXCL1 luc reporter activity was determined as described in the Materials and Methods (n = 4). (C) NMDFs were treated with IBOP for (a) 16 h and (b) 8 h. CXCL1 and CXCL8 (a) protein expression was determined through ELISA and (b) mRNA level was determined by RT-PCR and real-time PCR (n = 3).

We observed that CXCL1 and CXCL8 were highly expressed in the submucosal stroma of the CRSsNP specimens compared with those of the controls. Stromal fibroblasts–induced collagen deposition is crucial in tissue remodeling [8]. Moreover, the production of PGs mediated by COX commonly is essential in the inflammation process. Among these PGs, TXA_2_ is a proinflammatory mediator in several diseases, such as cardiovascular and kidney diseases [18]. Therefore, we tested whether U46619, a TXA_2_ analog, can affect CXC chemokine release in the CRSsNP-derived fibroblasts, namely NMDFs. The concentration- and time-dependent effects of U46619 on CXCL1 and CXCL8 secretions were examined in the NMDFs (Fig 2A). We observed that U46619 enhanced CXCL1 and CXCL8 secretion in a concentration-dependent manner, with 2μM U46619 being sufficient to cause the secretion of CXCL1 and CXCL8 (Panels a). Moreover, U46619 induced the CXCL1 and CXCL8 release in a time-dependent manner, and a slight increase was observed at 4 h of incubation and the maximal increase was observed after 16 h of incubation (Panels b).

To determine whether U46619 induces CXCL1 and CXCL8 mRNA expression, the NMDFs were treated with U46619 at indicated concentrations, following which the expression of CXCL1, CXCL8, and GAPDH mRNA was determined through RT-PCR and real-time PCR. In RT-PCR analysis, CXCL1 and CXCL8 mRNA expression was upregulated following 1μM U46619 stimulation and peaked thereafter (Fig 2B[a], left panels). In real-time PCR analysis, CXCL1 and CXCL8 mRNA expression was upregulated following 1μM U46619 stimulation and reached to peak at 2 μM of U46619, indicating that U46619 might affect the levels of CXCL1 and CXCL8 through transcriptional regulation (Fig 2B[a], right panels). To confirm this hypothesis, CXCL1 luciferase (luc) reporter construct was considered a representative and transfected to the NMDFs. The luc reporter activity was subsequently examined in the presence and absence of U46619. U46619 enhanced CXCL1 luc reporter activity in a concentration-dependent manner, suggesting the involvement of transcription regulation in CXC chemokine release in the NMDFs (Fig 2B[b]). Next, we investigated whether IBOP can cause a similar inductive effect on the NMDFs. Fig 2C depicts an increase of CXCL1 and CXCL8 secretion; the responses were concentration-dependent (Panels a). Furthermore, IBOP induced CXCL1 and CXCL8 mRNA expression in the NMDFs, as determined by RT-PCR and real-time PCR analysis (Panels b). These results confirm the involvement of TP receptor and transcriptional regulation in CXCL1 and CXCL8 induction.

### TP Expression in the Nasal Mucosal Specimens and NMDFs

**Fig 3:**
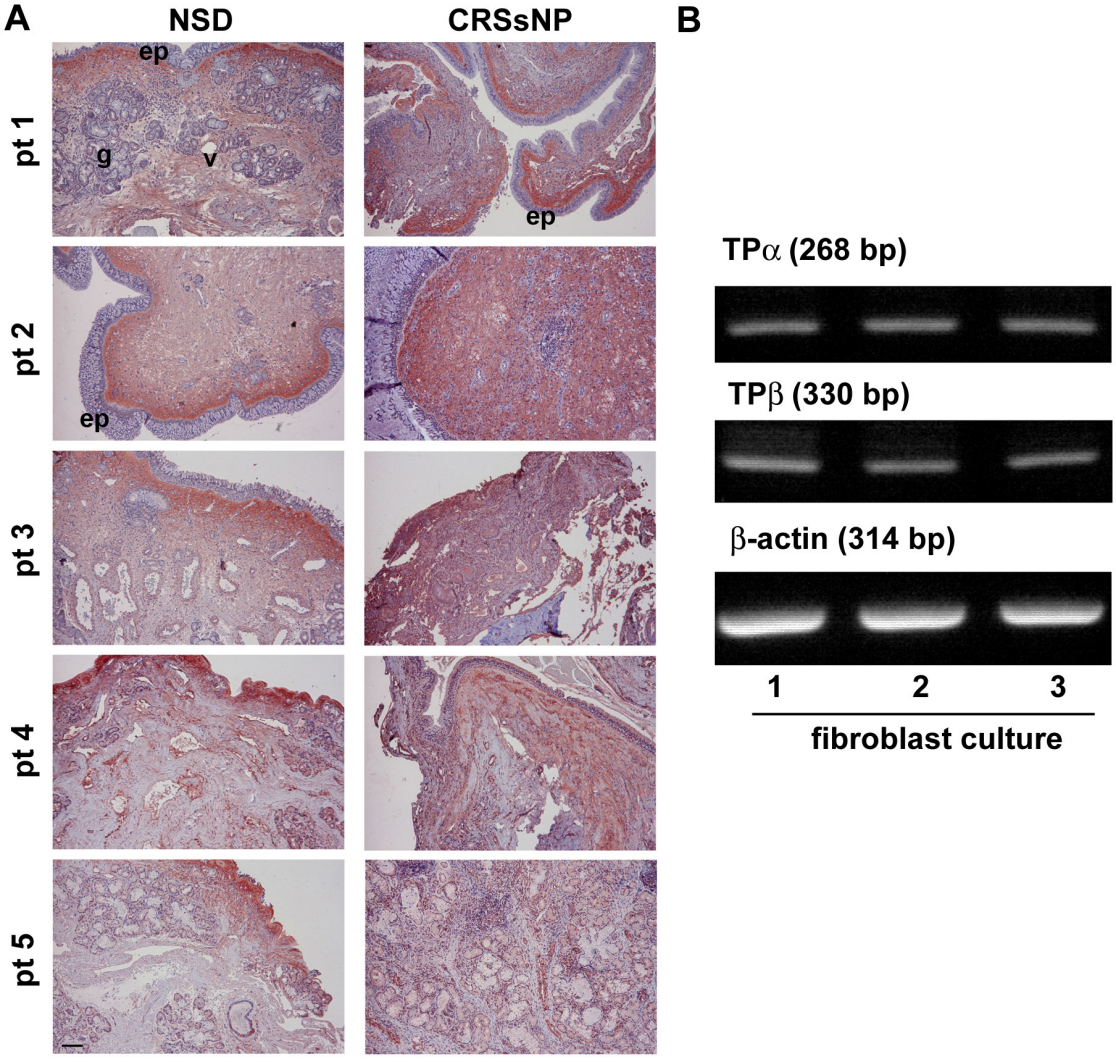
TP receptor expression in the CRSsNP mucosa and NMDFs. (A) Immunohistochemical staining of TP receptor distribution in the control and CRSsNP nasal mucosa. The inferior turbinates (control) and CRSsNP nasal mucosa from 5 individual patients (pt) were stained with the anti-TP receptor Ab. The immunoreactivity for TP receptor was predominantly found in the submucosal stroma of control and CRSsNP specimens. ep: epithelial cells; v: blood vessels; g: submucosal glands. Scale bar = 100 μm (B) TP receptor expression in the NMDFs. Total RNA was extracted from 3 NMDF cultures, and the expression of TPα, TPβ, and β-actin was analyzed through RT-PCR (n = 3).

We observed that U46619 and IBOP exhibited proinflammatory effects on the NMDFs (Fig 2). Therefore, determining the TP expression in CRSsNP tissues and control is crucial. In Fig 3A, immunohistochemical staining reveals that TP receptors were predominantly expressed in the submucosal area. The glands, vessels, and submucosal stroma considerably expressed the TP receptors both in the control and CRSsNP mucosa. TP staining in CRSsNP tissues did not significantly differ from that in the controls. TXA_2_ has been reported to signal through 2 TP isoforms, TPα and TPβ, in humans [23]. Therefore, the expressional level of TP receptor isoforms in the NMDFs was analyzed through RT-PCR. As shown in Fig 3B, TPα and TPβ mRNA were both expressed in 3 NMDF cultures with no apparent differences, indicating that both TPα and TPβ are expressed in the NMDFs.

### Effect of Signaling Inhibitors on U46619-Induced CXCL1 and CXCL8 Expression

**Fig 4:**
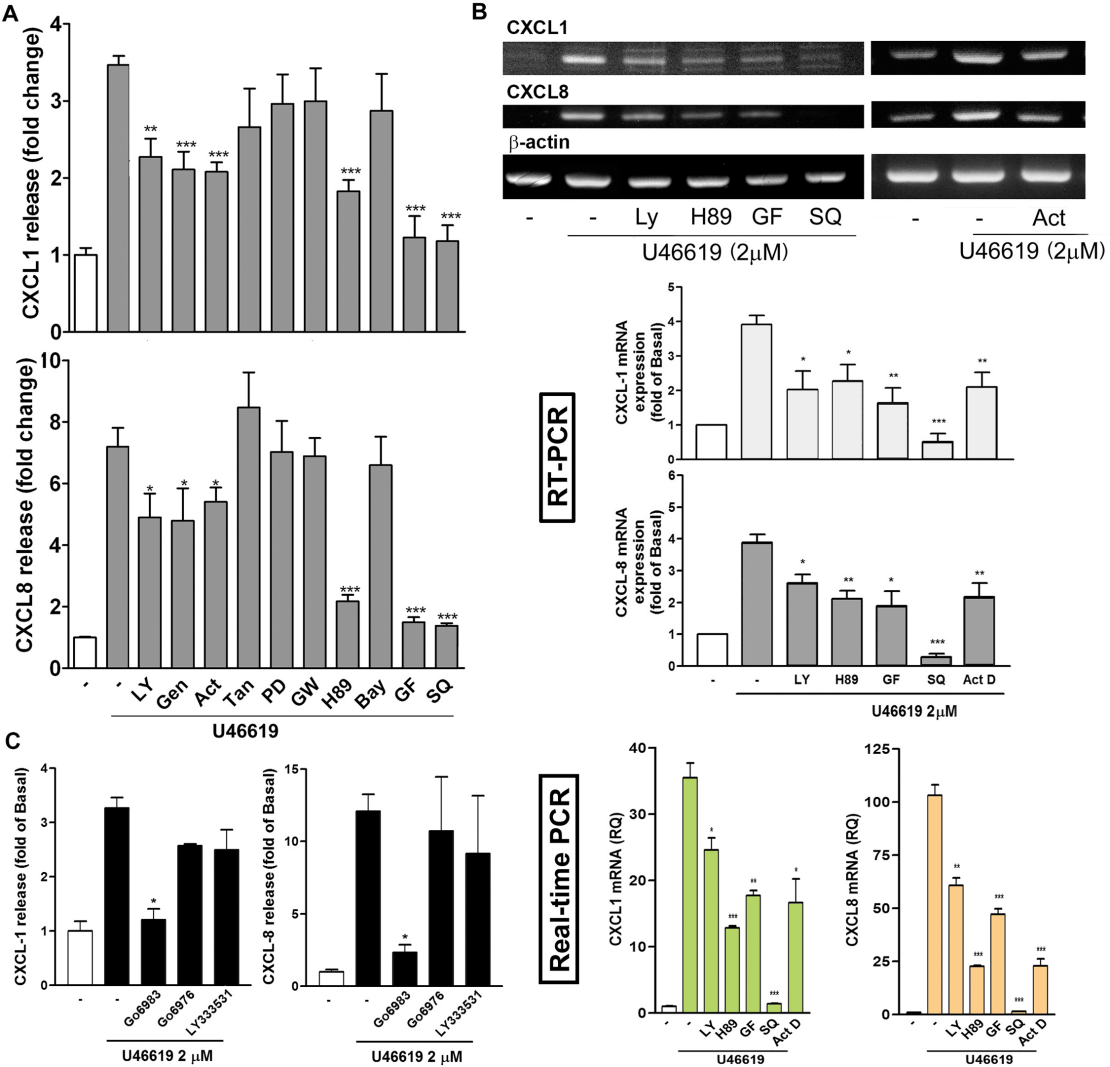
Effects of signaling inhibitors on U46619-induced CXCL1 and CXCL8 release and expression. NMDFs were pretreated with the indicated inhibitors for 30 min and followed by DMSO (-) or U46619 (2 μM) for (A and C) 16 h or (B) 4 h. CXCL1 and CXCL8 release in the culture media was analyzed through ELISA, and their mRNA expression was determined through RT-PCR and real-time PCR. The data are presented as mean ± SEM (n = 3–5). *P < 0.05, **P < 0.01, ***P < 0.001 versus control. LY: 10 μM; Gen: 10 μg/mL; Act D: 2 μM; Tan: 10 μM; PD: 10 μM; GW: 10 μM; H-89: 5 μM; Bay: 5 μM; GF: 2 μM; SQ: 10 μM.

We investigated the possible signaling pathways involved in the induction of CXCL1 and CXCL8 by using U46619. Several signaling inhibitors targeting PI-3K, protein kinases, NF-κB signaling pathway, transcription, and protein translation were used in this assay. Fig 4A shows that the PI-3K inhibitor (LY294002), tyrosine kinase inhibitor (genistein), DNA transcription inhibitor (Act. D), PKA inhibitor (H89), PKC inhibitor (GF109203X), and TP receptor antagonist (SQ29548) significantly inhibited CXCL1 and CXCL8 release through U46619 stimulation; however, the AP-1 transcription factor inhibitor (tanshinone IIA), MAPKK inhibitor (PD98059), PPARγ inhibitor (GW9662), and NF-κB inhibitor (BAY117085), had no effect. The MTT assay revealed that the inhibition was not caused by a decrease in cell viability (data not shown).

We subsequently examined whether the PI-3K inhibitor (LY294002), DNA transcription inhibitor (Act. D), PKA inhibitor (H-89), PKC inhibitor (GF109203X) and TP receptor antagonist (SQ29548) affected CXCL1 and CXCL8 mRNA expression because they inhibited U46619-induced CXCL1/8 release. The inhibitors all inhibited U46619-induced CXCL1 and CXCL8 mRNA expression (Fig 4B). The data indicate that TP receptor-, PKA-, PKC- and PI-3K-related signaling pathways might mediate U46619-induced CXCL1 and CXCL8 mRNA transcription.

Next, we determined the type of PKC participating in CXCL1/8 induction because several PKC isoforms exist [24,25]. Go6976, an inhibitor more specific to PKC α/β, and LY333531, a selective and potent inhibitor of PKCβ1/β2, did not inhibit U46619-induced CXCL1 and CXCL8 expression (Fig 4C). By contrast, Go6983, a pan-PKC inhibitor, markedly inhibited U46619-induced CXCL1/8 expression, suggesting that the PKCs other than PKCα and β mediate CXCL1/8 induction.

### Activation of ERK, PI-3K, PKA, PKC, and CREB on U46619 Treatment

**Fig 5:**
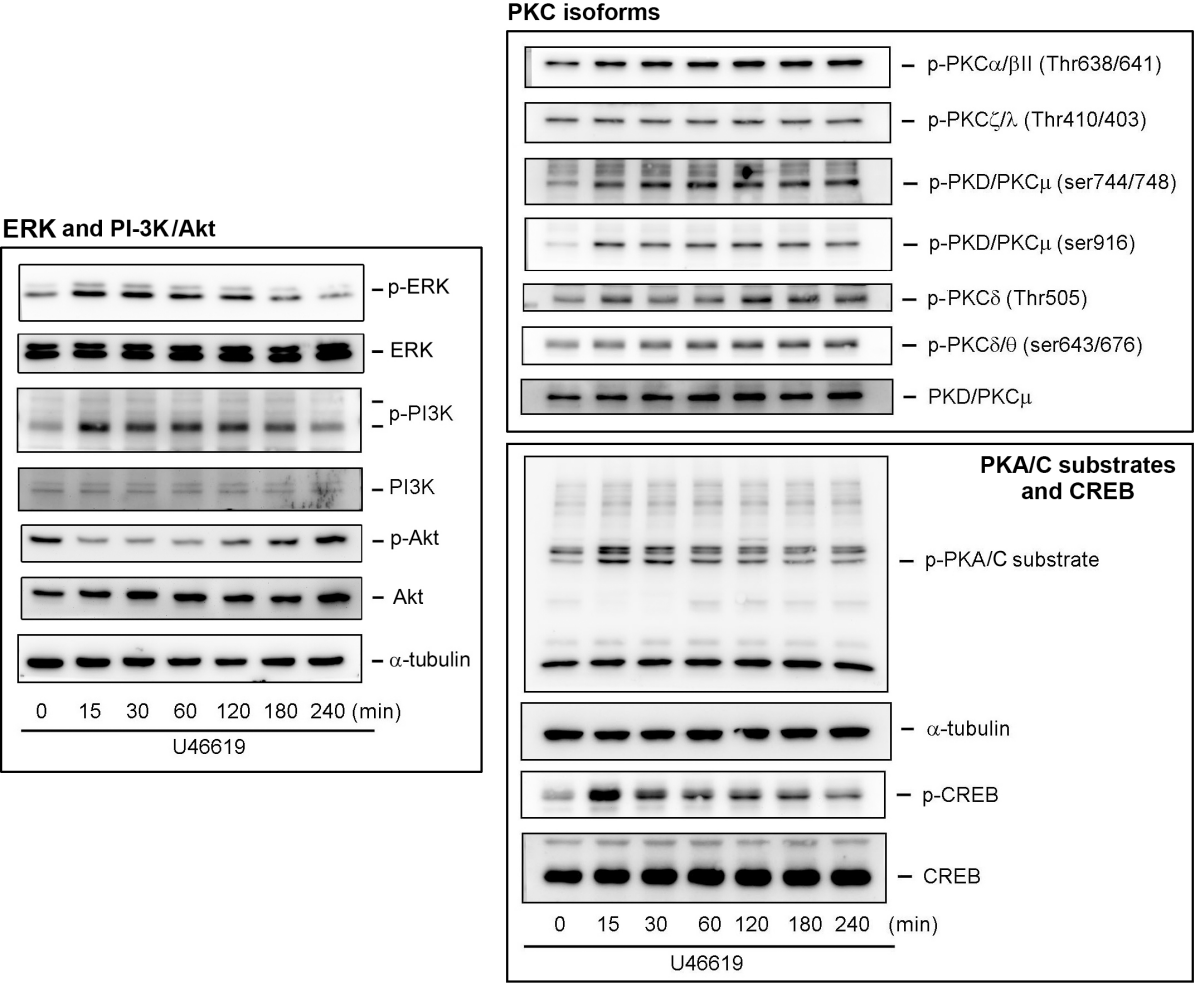
U46619 induces ERK, PI-3K, PKC, PKA/C substrates, and CREB activation. NMDFs were treated with U46619 (2 μM) for the indicated time intervals. After incubation, the cell lysates were analyzed through Western blotting (n = 5–7).

Fig 4 shows that the PI-3K, PKA, and PKC inhibitors suppressed U46619-induced CXCL1 and CXCL8 release. We subsequently investigated whether U46619 can directly activate the corresponding signaling pathways in the NMDFs. Fig 5 shows that U46619 considerably activated (phosphorylated) PI-3K in the NMDFs in a time-dependent manner. However, its downstream Akt did not appear to be activated because a higher basal level. Although the MAPKK inhibitor (PD98059) did not affect U46619-induced CXCL1 and CXCL8 release (Fig 4A), ERK was similarly activated to PI-3K. In addition, the PKA and PKC signaling pathways, including PKC isoforms, PKA/C substrates, and their possible downstream nuclear transcription factor (CREB), were investigated in the NMDFs. Fig 5 also clarifies that the phosphorylation of the PKA/C substrates and CREB was detected at 15-min following U46619 stimulation and could be sustainably activated thereafter. The phosphorylation of PKCα/βII, PKD/PKCμ, and PKCδ/θ were observed, whereas the PKCζ/λ remained unchanged, suggesting that PKA, PKC, and possibly their downstream transcription factor CREB are involved in the TXA_2_-TP receptor-mediated signal transduction pathway.

### Signaling Context of PI-3K, PKA/C, and CREB Activation in U44619-Induced Signaling

**Fig 6:**
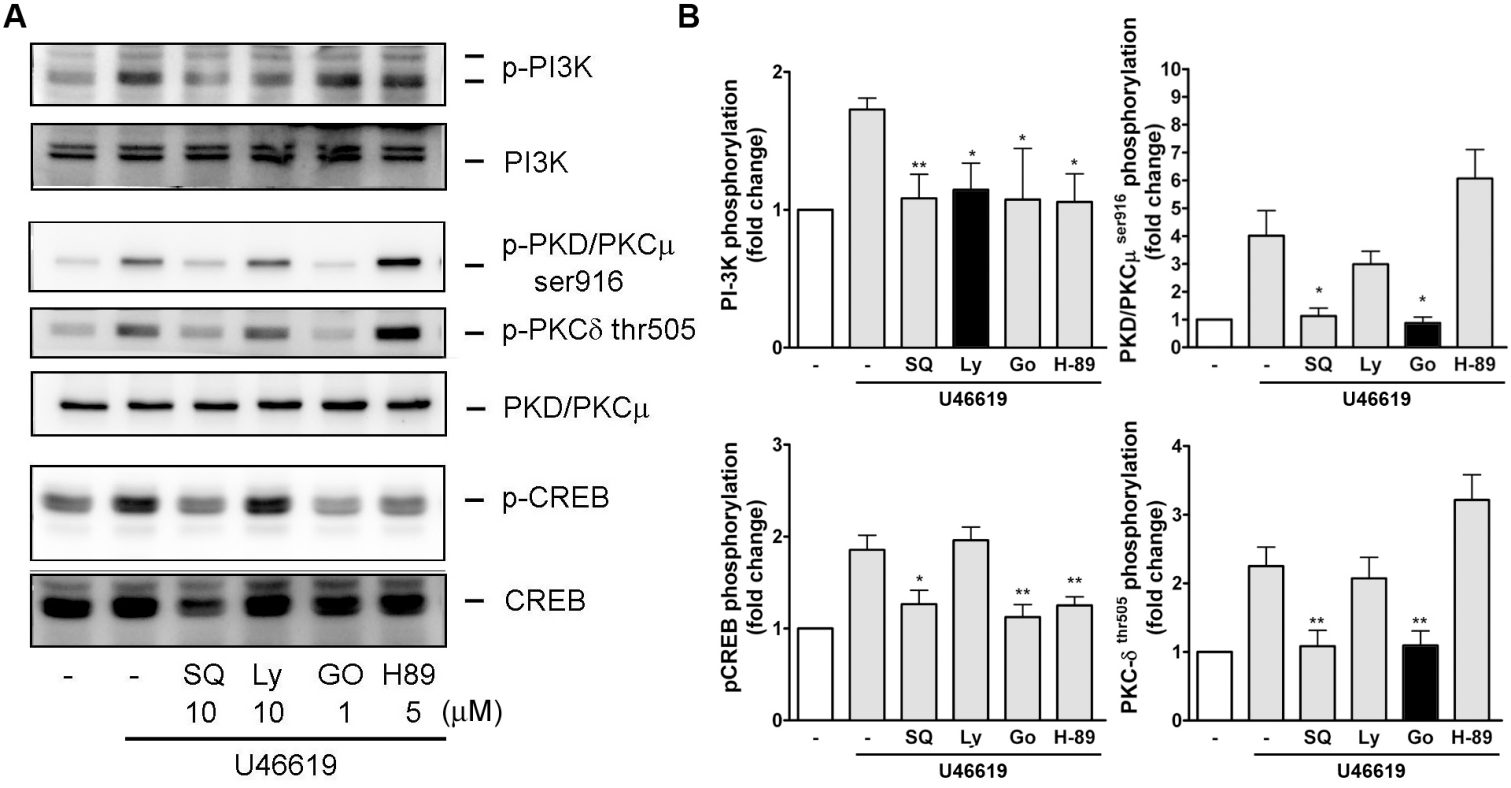
Relationship of PI-3K, PKC, and CREB activation in U44619-induced signaling. NMDFs were pretreated with SQ29458 (SQ, 10μM), LY294002 (LY, 10 μM), Go6983 (Go, 2 μM), and H-89 (5 μM) for 30 min and followed by stimulation with U46619 (2 μM) for 15 min. (A) A representative blot. (B) Similar results were obtained through densitometry. Data are expressed as mean ± SEM (n = 5–8). *P < 0.05, **P < 0.01 versus U46619 alone.

Western blotting was performed in the presence of the specific pharmacological inhibitor to understand the activation relationship of these kinases in response to U46619 in the NMDFs. The effectiveness of TP receptor antagonist (SQ29548), PI-3K inhibitor (LY294002), pan PKC inhibitor (Go6983), and PKA inhibitor (H-89) on their relevant kinases was evaluated, which revealed complete inhibition at the concentration used (blots and black bars in Fig 6A and 6B). LY294002 (PI-3K inhibitor) did not suppress the U46619-induced CREB and PKC activation. However, Go6983 (a pan-PKC inhibitor) inhibited U46619-induced PKCδ/θ, PKD/PKCμ, PI-3K, and CREB activation. Moreover, H-89 (PKA inhibitor) inhibited the activation of PI-3K and CREB but had no effect on the activation of PKC. Therefore, PKA and PKC partly affected PI-3K activation in the NMDF in response to U46619. Go6986 and H-89 both suppressed the CREB activation, suggesting that PKC and PKA were activated before CREB in the NMDFs stimulated by U46619. These findings suggest that U46619 can simultaneously and independently activate PKA, PKC, and PI-3K. Following activation, PKA and PKC activate PKA/C substrates and potentiate CREB phosphorylation and crosstalk with PI-3K.

### Role of cAMP and PKA in U46619-Induced CXCL1/8 Expression

**Fig 7:**
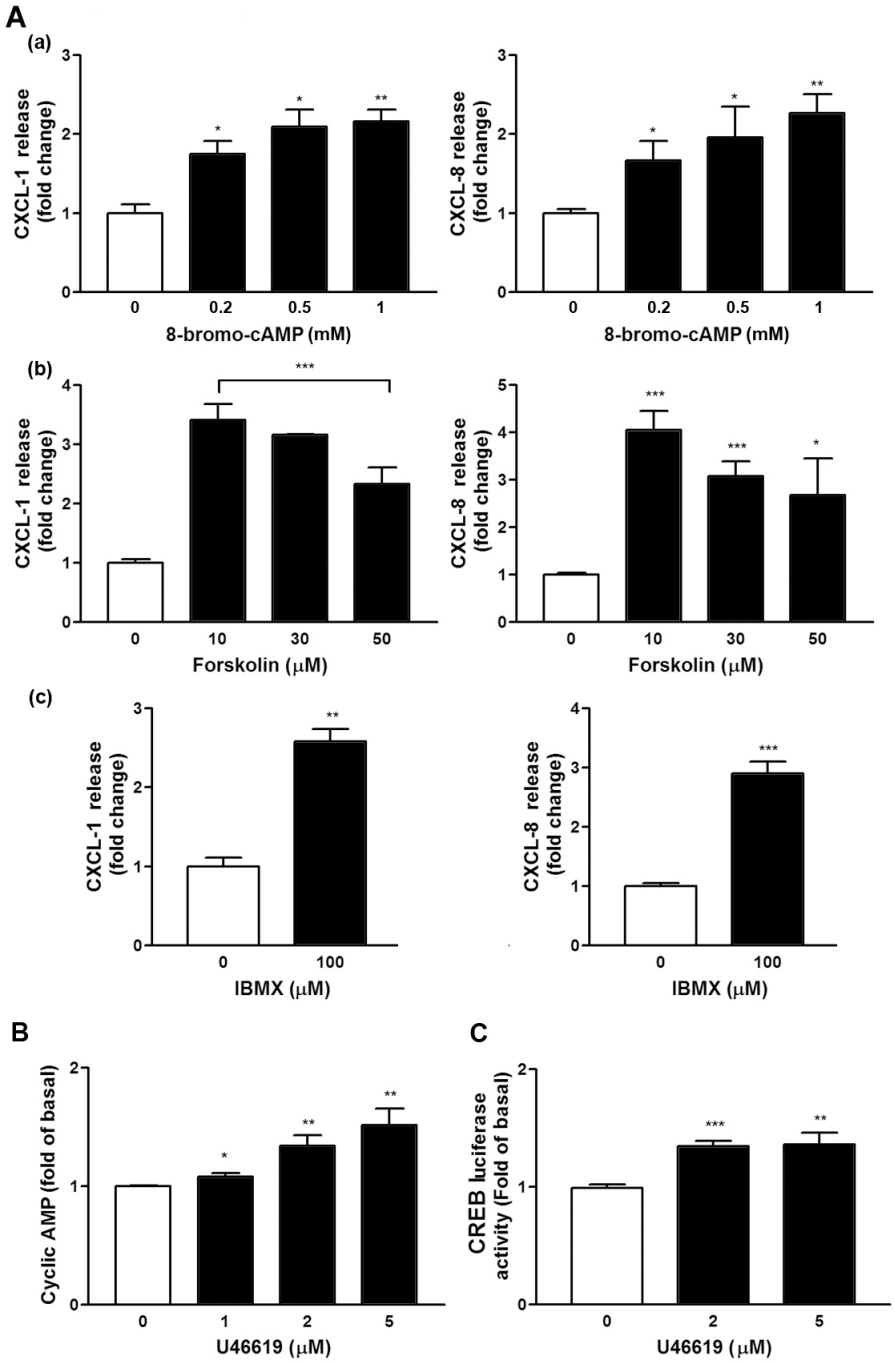
Effect of cAMP/PKA-related compounds on CXCL1/8 induction and U46619 on intracellular cAMP production and CREB luc reporter activity. (A) NMDFs were treated with the indicated compounds for 16 h, and CXCL1 and CXCL8 in the culture medium were analyzed through ELISA. (B) NMDFs were treated with U46619 for 10 min, and intracellular cAMP was assayed through ELISA (n = 4). (C) NMDFs were transfected with the CREB promoter luc reporter for 48 h and followed by U46619 stimulation. CREB luc reporter activity was determined as described in the Materials and Methods (n = 4).*P < 0.05, **P < 0.01 and ***P < 0.001 versus control.

Several cAMP and PKA-activating compounds were used to verify the importance of cAMP/PKA pathway in inducing CXCL1 and CXCL8 in the NMDFs. Fig 7 shows that 8-bromo-cAMP, a cell permeable cAMP analog that activates cAMP-dependent PKA, induced CXCL1 and CXCL8 expression. Moreover, forskolin, a labdane diterpene produced by the Indian Coleus plant (*Coleus forskohlii*), which activates the enzyme adenylyl cyclase and increases intracellular levels of cAMP, and IBMX (3-isobutyl-1-methylxanthine), a phosphodiesterase inhibitor, were directly applied to the cells. All of them promoted the release of CXCL1 and CXCL8, suggesting that cAMP-PKA axis enhances CXC chemokine production by U46619 in the NMDFs. Next, we examined whether U46619 is capable of triggering cAMP production. Fig 7B depicts that U46619 concentration-dependently increased intracellular cAMP production. In line with these observations, transfection of CREB luc reporter to the NMDFs followed by U46619 treatment (2 and 5 μM) increased the CREB luc reporter activity.

### Involvement of PKCμ/PKD and CREB in U44619-Induced CXCL1 and CXCL8 Induction

**Fig 8:**
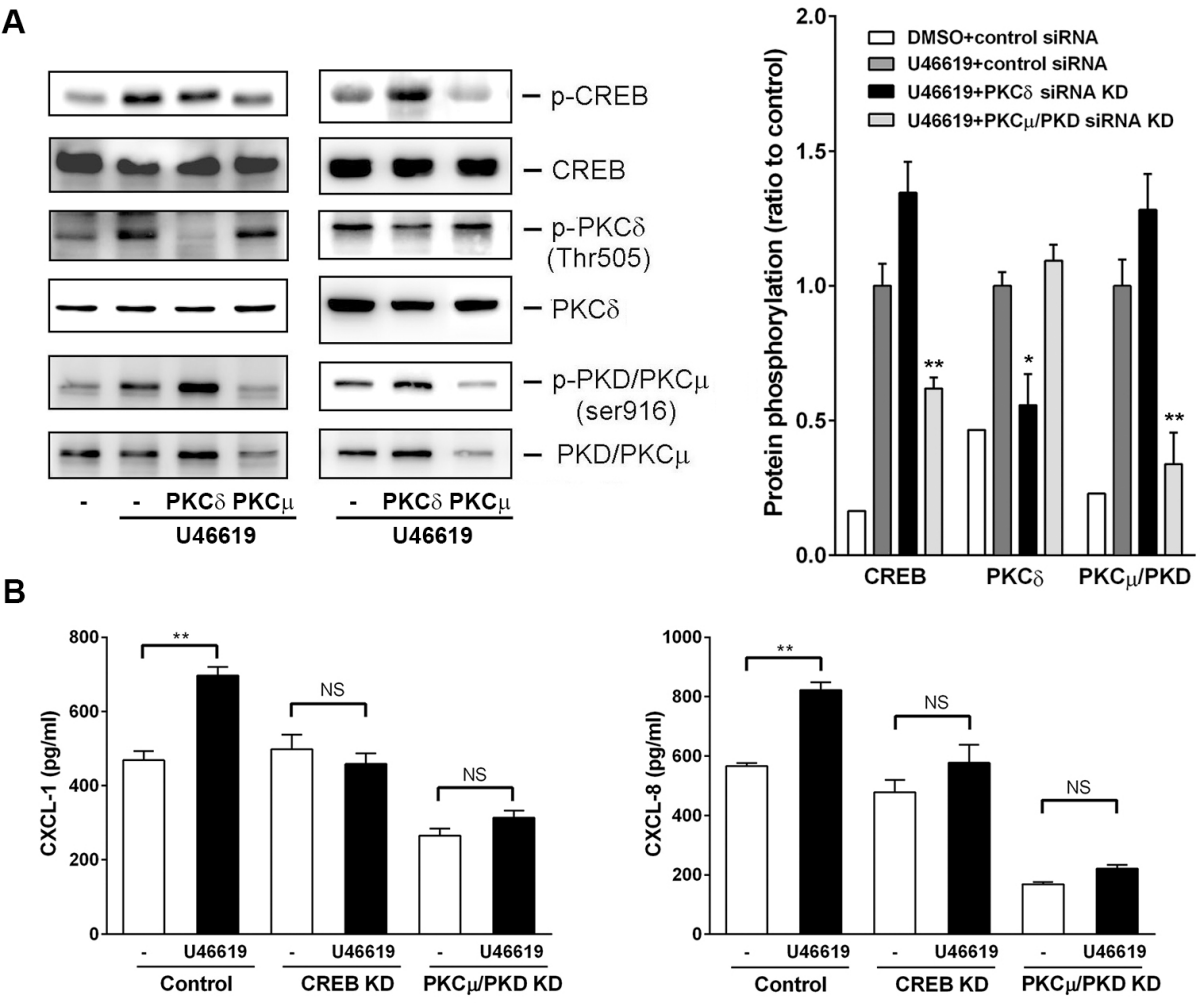
Effect of CREB, PKCδ, and PKCμ/PKD KD on U46619-induced signaling and CXCL1/8 expression. (A) Effect of PKCδ/θ and PKCμ/PKD KD on U46619-induced signaling: Cells were transfected with control or PKCδ and PKCμ/PKD siRNA for 72 h and followed by addition of vehicle or U46619 (2 μM) for 120 min. Protein expression and phosphorylation was determined through Western blotting. Two representative results were shown from four independent results. (B) Effect of PKCμ/PKD or CREB KD on U46619-induced CXCL1/8 expression: Cells were transfected with control, PKCμ/PKD, or CREB siRNA for 72 h and followed by addition of vehicle or U46619 (2 μM) for 16 h. CXCL1 and CXCL8 expression was determined through ELISA (n = 3–4). *P < 0.05, **P < 0.01 versus control.

We demonstrated that PKD/PKCμ and PKCδ/θ were possibly involved in U46619-induced CXCL1 and CXCL8 induction in the NMDFs. Next, we determined the PKC isoforms responsible for this induction. Small interference RNAs (siRNAs) were introduced to the knockdown (KD) of PKD/PKCμ and PKCδ, respectively. Fig 8 depicts transfection of PKD/PKCμ and PKCδ siRNAs to the NMDFs, which led to a marked decrease in expression level of PKD/PKCμ but a slight decrease in that of PKCδ. However, an apparent reduction in phosphorylation was observed in each respective PKC isoform (PKD/PKCμ and PKCδ). Surprisingly, only PKD/PKCμ siRNA KD significantly reduced U46619-induced CREB phosphorylation. Consistent with this finding, PKD/PKCμ and CREB siRNA KD suppressed U46619-induced CXCL1 and CXCL8 release in the NMDFs (Fig 8B).

## Discussion

Arachidonic acid metabolites control crucial cellular processes, including cell proliferation, apoptosis, metabolism, and migration [26]. TXA_2_ is one of the active arachidonic acid metabolites and is a potent mediator involved in allergic rhinitis [4]. TXA_2_ receptor α (TPα) expression has been observed in the nasal mucosa from patients with nasal obstruction refractory to medical treatment [4]. In addition, TXA_2_ receptor mRNA was detected both in the nasal inferior turbinates with allergic rhinitis and in the maxillary sinus mucosa with chronic inflammation [27]. In this study, we demonstrated that TP receptors were expressed in the nasal mucosa either from patients with nasal septal deviation or CRSsNP. We did not observe considerable differences between the staining of the CRSsNP and control groups. This is consistent with the study conducted by Maesako et al. [27], revealing that the mRNA of TXA_2_ receptor was expressed at a similar level in the nasal inferior turbinates with allergic rhinitis and in the maxillary sinus mucosa with chronic inflammation [27]. Moreover, we observed that both TPα and TPβ were expressed in the NMDFs (Fig 3B). These results implicate a possible role of TP receptor and TXA_2_ in physiological and pathophysiological conditions.

One of the crucial findings of this study was the mechanism underlying CXCL1 and CXCL8 induction by TXA_2_. Recent studies on the mechanism of lung cancer invasion have reported that TXA_2_ possibly acts through TPα and subsequently transactivates protease-activated receptor 2, finally causing CCL2 chemokine expression, which potentiates the macrophage for tumor invasion [28]. In addition, TXA_2_ induces NF-κB activation and increases the endothelial permeability through IL8 (CXCL8) induction [29]. Our results revealed that U46619 regulated the CXC chemokine release in the NMDFs through the TP receptor because the induction was approximately abolished completely by SQ29458 (a TP receptor antagonist). Moreover, the speculation arises from the observations that the NMDFs expressed both TPα and β receptors and the TP receptor agonist, IBOP, induced CXCL1 and CXCL8 mRNA and protein expression in the NMDFs similar to U46619 (Figs 2C and 3B). Furthermore, we demonstrated that following U46619 treatment, the activation of TP receptor not only activated PI-3K signaling but also independently activated the PKA and PKC signaling pathways (Figs 5–8). This indicates that TXA_2_ acts as a stimulator and can induce several signaling pathways in the NMDFs.

The regulation at a transcriptional level of CXCL1 and CXCL8 following U46619 stimulation was demonstrated by the following findings. First, U46619 and IBOP enhanced CXCL1 and CXCL8 mRNA transcription following U46619 stimulation (Fig 2B and 2C). Second, Act. D, a gene transcription inhibitor, attenuated U46619-induced CXCL1/8 protein release and mRNA expression (Fig 4); regarding cellular kinases responsible for the transcriptional regulation in U46619-induced CXCL1/8 release in the NMDFs from the CRSsNP mucosa, we suggest the involvement of PI-3K, PKA/C, and CREB. Consistent with these findings, U46619 directly activated the corresponding cellular signaling components, including PI-3K, PKC isoforms (PKCα/βII, PKD/PKCμ, and PKCδ), PKA/C substrates, and CREB (Fig 5). Their activation was affirmed by using pharmacological inhibitors, permeable activators, and siRNA KD (Figs 6–8). The PI-3K inhibitor did not suppress CREB and PKA/C activation (Fig 6), suggesting that PI-3K and PKA/C may be activated independently by U46619. Moreover, because PKC and PKA/C substrates were activated and the inhibitors for PKC and PKA compromised PI-3K and CREB phosphorylation, we suggest that both PKA and PKC can activate CREB and might crosstalk with PI-3K/JNK pathway for U46619-mediated CXCL1 and CXCL8 induction (Fig 5). This hypothesis is supported by the following evidence. First, 8-bromo-cAMP (a permeable cAMP more resistant to PDE hydrolysis), forskolin, and IBMX (a PDE inhibitor) can directly induce CXCL1 and CXCL8 mRNA/protein expression (Fig 7A). In addition, U46619 increased intracellular cAMP production and enhanced CREB luc reporter activity (Fig 7B and 7C). The siRNA KD assay further confirmed the critical role of PKCμ/PKD but not PKC δin CREB activation and CXCL1/8 induction (Fig 8).

PI-3K/Akt is predominantly considered a survival player for cellular proliferation and survival. We previously revealed that the JNK-PI-3K-AKT signaling pathway is involved in TNF-α–induced CXCL1 expression in pulmonary epithelial cells [30]. However, in the current study, the Akt was not activated following U46619 stimulation (Fig 5). Therefore, a downstream substrate for PI-3K remains to be further determined. This pathway was not revealed to be a major pathway because the PKC or PKA inhibition caused more reduction of CXCL1/8 release compared with PI-3K inhibition (Fig 4).

IL-1β has been reported to induce CXC chemokine gene expression in NSCLC A549 cells, which participates in the angiogenic procedure of tumorigenesis. This process is modulated by the CREB [31]. In addition, CXCL8 is crucial in cystic fibrosis, a neutrophil dominant inflammatory disease. When encountering a suitable stimulus, the *cxcl8* gene is transcriptionally regulated by the following transcription factors: NF-κB, activating protein (AP-1), CAAT/enhancer-binding protein β (C/EBPβ, also known as NF-IL-6), C/EBP homologous protein (CHOP) and CREB [32]. Therefore, the CXCL8 level and these transcription factors are crucial for treating cystic fibrosis. Moreover, similar to cystic fibrosis, CRSsNP is characterized as a neutrophil-skewed inflammatory disease. Thus, controlling the chemokine release and neutrophil activation is critical in CRSsNP.

**Fig 9:**
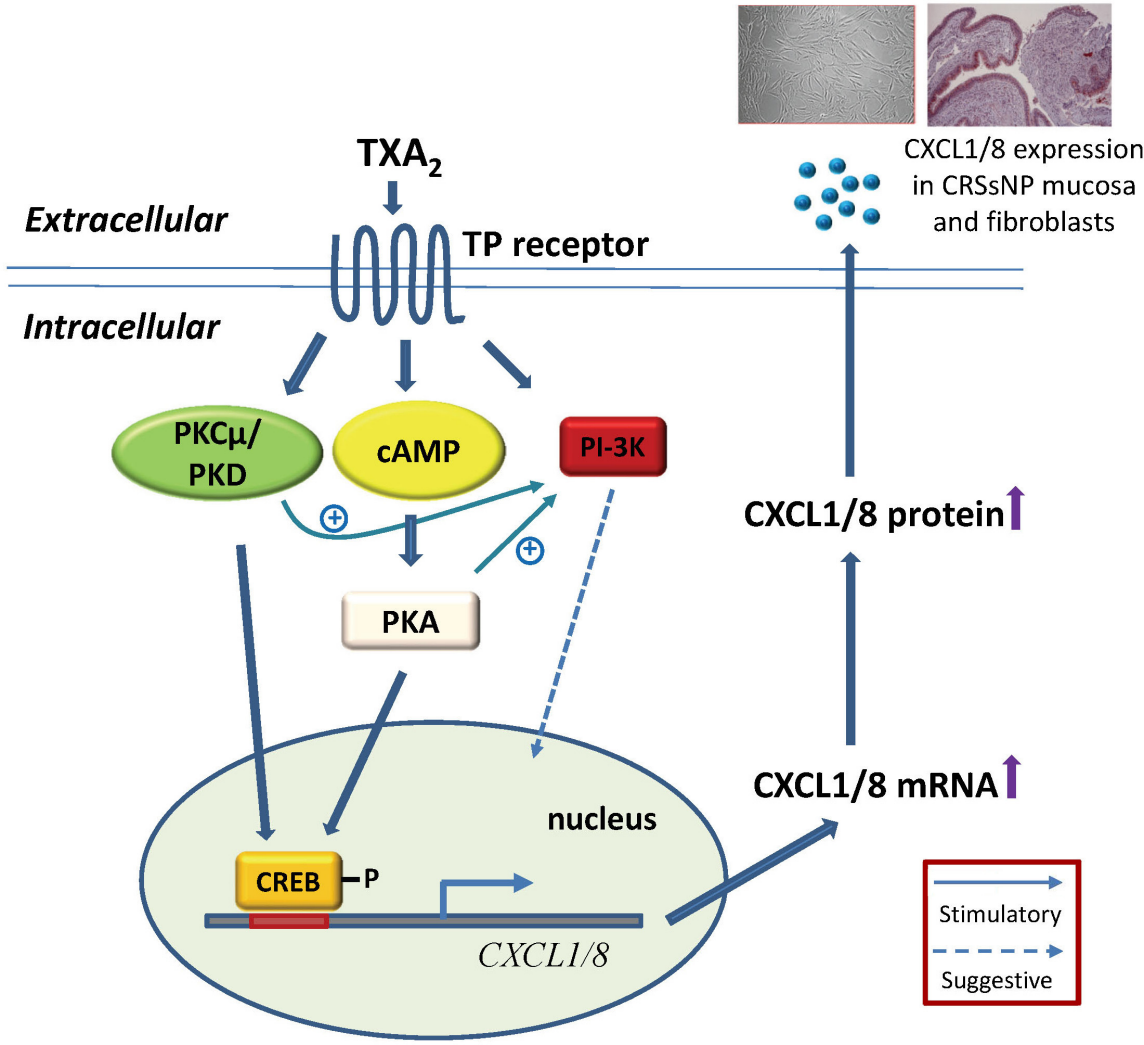
Proposed mechanism of TXA2-induced CXC chemokine expression in NMDFs.

In conclusion, we demonstrated that U46619 can induce CXCL1/8 mRNA and protein expression in NMDFs through PI-3K, cAMP/PKA, PKCμ/PKD, and CREB-dependent signaling pathways. Fig 9 illustrates that TXA_2_ stimulation simultaneously activated PI-3K, PKA, and PKCμ/PKD. cAMP/PKA and PKCμ/PKD activation lead to CREB phosphorylation and crosstalk with PI-3K pathway, causing CXCL1/8 mRNA and protein expression. Furthermore, we demonstrated that CXCL1 and CXCL8 are highly expressed in the nasal mucosa of CRSsNP, whereas the TP receptor for thromboxane A_2_ are abundantly expressed both in the control and CRSsNP mucosa. Our study elucidates the underlying mechanism and provides the first evidence for thromboxane A_2_ in inducing CXCL1 and CXCL8 chemokine in CRSsNP-derived nasal fibroblasts.
